# Skin surface debris as an archive of environmental traces: an investigation through the naked eye, episcopic microscope, ED-XRF, and SEM–EDX

**DOI:** 10.1007/s00414-023-03021-1

**Published:** 2023-05-25

**Authors:** Vera Merelli, Giulia Caccia, Debora Mazzarelli, Lorenzo Franceschetti, Orlando Paciello, Letizia Bonizzoni, Marco Caccianiga, Carlo Campobasso, Cristina Cattaneo

**Affiliations:** 1 Labanof (Laboratorio Di Antropologia E Odontologia Forense), Sezione Di Medicina Legale, Dipartimento Di Scienze Biomediche Per La Salute, Università Degli Studi Di Milano, Milan, Italy; 2 Dipartimento Di Medicina Sperimentale, Università Degli Studi Della Campania Luigi Vanvitelli, Naples, Italy; 3 Dipartimento Di Medicina Veterinaria E Produzioni Animali, Università Degli Studi Di Napoli Federico II, Naples, Italy; 4 Dipartimento Di Fisica Aldo Pontremoli, Università Degli Studi Di Milano, Milan, Italy; 5 Dipartimento Di Bioscienze, Università Degli Studi Di Milano, Milan, Italy

**Keywords:** Crime scene, Debris, External examination, ED-XRF spectroscopy, SEM–EDX analysis, Episcopic microscope

## Abstract

Traces from bodies can be of various nature, for example of biological or inorganic origin. Some of these historically have received more consideration than others in forensic practice. Samplings of gunshot residues or biological fluid traces are commonly standardized, whereas macroscopically invisible environmental traces are usually ignored. This paper simulated the interaction between a cadaver and a crime scene by placing skin samples on the ground of five different workplaces and inside the trunk of a car. Traces on samples were then investigated through different approaches: the naked eye, episcopic microscope, Scanning Electron Microscopy (SEM) with Energy-dispersive X-ray spectroscopy (EDX) and Energy Dispersive X-Ray Fluorescence (ED-XRF). The purpose is to provide the forensic scientist with the awareness of the value of debris on skin and then to highlight implications for forensic investigations. Results demonstrated that even naked eye observation can reveal useful trace materials, for defining the possible surrounding environment. As a next step, the episcopic microscope can increase the number of visible particulates and their analysis. In parallel, the ED-XRF spectroscopy can be useful to add a first chemical composition to the morphological data. Finally, the SEM–EDX analysis on small samples can provide the greatest morphological detail and the most complete chemical analysis, although limited, like the previous technique, to inorganic matrices. The analysis of debris on the skin, even with the difficulties due to the presence of contaminants, can provide information on the environments involved in criminal events that can add to the investigation framework.

**Supplementary Information:**

The online version contains supplementary material available at 10.1007/s00414-023-03021-1.

## Introduction

When investigating a violent crime, one crucial question is under what circumstances the victim died. To this end, the external examination of the body is mandatory and can provide key information. Several types of materials or traces can be collected from a corpse: for example biological of human origin (i.e. blood, sperm, saliva), biological of nonhuman origin (i.e. traces of botanical, fungal, or animal origin), inorganic (i.e. varnishes, gunshot residues) and traces of soil or minerals. Although the analysis of all these trace materials collected on the body may provide circumstantial evidence(s) [1, 2], not all of them receive equal consideration in the medicolegal context. Those of human origin are one of the main objects of interest and are universally included and standardised in all protocols of approach to the body (at least in the case of well-preserved bodies); instead, the others are often neglected, and their management is left to the discernment of the individual operator [3–5]. Nevertheless, the fact that the environment leaves macroscopically visible or invisible traces on the victim is not in dispute [6, 7], and, in some cases, the macroscopically invisible trace could be the only trace of the environment where a crime occurred. The need arose from homicide cases that the authors dealt with in recent years, the most significant of which concerns the partially skeletonized corpse of a girl found in a field. In that case, several elements suggested that the victim may have been killed elsewhere and subsequently deposited where the discovery occurred. For this purpose, several investigations aimed at studying the interaction between the corpse and the environment were carried out. Skin and clothing samples were taken upon autopsy examination and subjected to SEM/EDX analysis. Samples revealed the widespread presence of micro-traces rich in calcium powders (calcium oxide, CaO) and numerous metallic spheres made of iron (Fe), chromium (Cr), and/ or nickel (Ni). The source of such material was sought in all the environments usually visited by the victim without finding matches. So, a forensic geologist and a mechanical engineer were requested. They direct the research toward an environment connected with the construction industry; this element allowed the identification of the main suspect for the homicide [8]:

Building on this experience, the present work aims at reconstructing the interaction between a cadaver and six different environments simulating a hypothetic crime scene: skin samples were placed in five different workplaces and inside the trunk of a car. The samples were then studied using four methods of investigation: a morphological one with the naked eye, with an episcopic microscope, and through Scanning Electron Microscopy (SEM) and a spectroscopic approach by Energy-dispersive X-ray spectroscopy (EDX) and Energy Dispersive X-Ray Fluorescence (ED-XRF). Both SEM–EDX and ED-XRF are well-known techniques for the search of information for the analysis of GSR (gunshot residues) on both skin and garments [5, 9–14] or to identify the material of a weapon [15–19], but rarely used to pinpoint a victim's environment of origin or passage.

Hence the goal of the present study is to verify, as done previously for several inorganic substrates [20–25], if and how skin could be the recipient of traces from the environment and highlight the importance of corpse debris research to identify where the body has been exposed or what it has been put in direct contact with and to evaluate any qualitative changes of the debris over time, with particular attention to the progression of putrefactive phenomena.

### Materials and Methods

A swine skin was designated as the optimal source for producing the samples, due to its similarity with human skin [26]. The skin was obtained from an animal that died from causes unrelated to this study and, immediately after recovery, was stored in a freezer at -20 °C. Half of its surface was shaved. The entire skin was thoroughly rinsed with water and then cut in 122 squares measuring 3 × 3 cm each.

Six sites were selected for experiments reported in this paper: a florist, a bakery, a carpentry, a turnery, a trunk of a car, and a construction site. The following samples were placed in each experimental environment:

- Two squares, one hairy (H +) and other one hairless (H-), were put in direct contact with a plane surface of the environment (floor or tables, shelves) by adhering the upper surface of the skin sample for 30 s.
- Two squares, one hairy (H +) and other one hairless (H-), were exposed to the open air in the same environments for 4 h; in this case, the sample was placed on a plane with the upper surface exposed to the air.

The following control samples were provided:

- One hairy square (H +) and one hairless (H-) underwent the same analysis intended for studying the other samples without being exposed to the environment.
- One graphite stub (suitable for direct analysis by SEM–EDX because it is electrically conductive) was exposed to the environment for 4 h and one put in direct contact with the same plane surface selected for the test samples, for 30 s, and then analyzed by SEM–EDX.

Therefore, the number of squares was 4 for each of the six experimental environments and for each of the five experimental times, given the destructive nature of some techniques. The first set of 24 samples was studied immediately after the 4 h of exposure and after the direct contact with the environment (Time 0); the remaining samples were stored in plastic boxes open but protected from light with paper covers in a controlled environment. New sample analyses were carried out on the stored samples after 1 day (Time 1), after 1 week (Time 2), after 2 weeks (Time 3), and after 1 month (Time 4). Total skin samples studied was 122 [(4 × 6 (experimental environment) × 5 (experimental time)) + 2 (negative control samples)].

Each square, comprising the two negative controls, underwent four subsequent analytical processes:

- naked eye observation
- episcopic microscope observation (Leica Wild Heerbrugg Op Mikroskop M650)
- double direct ED-XRF study on the upper half (Assing Lithos System)
- SEM–EDX study of the lower half after application and graphite metallization of two 0.5 cm.^2^ graphite tapes (Cambridge Stereoscan 360)

The operating parameters and analytical methodologies applied with the different instruments are the following:

Naked eye—All samples were observed without instrumentation, photographed, and all material of interest was classified and recorded.

Episcopic microscope—Each sample was visually traversed twice following parallel lines at 16X and 25X magnifications from top to bottom. All material of interest was documented photographically and classified during this observation.

ED-XRF—The instrument was equipped with a low power X-ray tube with Mo anode and a zirconium transmission filter (100 µm) producing a monochromatic excitation spectrum. The working conditions were 25 kV and 0.3 mA. All samples were subjected to two analyses. Each analysis generates a graph showing the peaks corresponding to the chemical elements most represented in the investigated area, which corresponds to a surface of about 0.5 cm^2^ of skin.

SEM–EDX—The instrument has been equipped with lanthanum LaB6 processed filament, the acceleration voltage is set at 20 le and the working distance at 25 mm. Each sample is entirely explored at magnifications of 500X for a general overview and then specific elements were analyzed with magnifications up to 300.000X. All material of interest is documented photographically and through spectroscopic analysis, counted, and classified during this observation.

All the elements found with the different techniques in the environmental positive control samples (graphite stubs) of the six environments tested were assigned a value from 0 to 5, this score was produced by subtracting from 6 (total number of environments) a point for each environment that shares this element. This value represents the specificity score of these elements: score 0 if the element is present in every environment, and therefore useless for identifying or excluding any of them, score 1 if the element is present in 5 environments, so only valuable for excluding 1 of the 6, score 2 if the element is present in 4 environments, so only valuable for excluding 2 of the 6, score 3 if the element is present in 3 environments, and, with the same principle, score 4 if present in 2 environments and 5 if present in only in 1 environment. Five represents the maximum score and identifies elements exclusive to a single environment and therefore highly identifying. In this way, each environment's score was calculated for both morphological and chemical elements; a higher total score will therefore be given to environments (through the score obtained from the graphite samples used as positive control) with a greater number of elements and with exclusive elements. Similarly, skin samples were scored. In this way, it was possible to assess a score for each sample (denoted as *value*) and then compare that score with those obtained by the control sample from the same environment (denoted as *tot*). With the same criterion, scores were attributed to elements not present in the control samples. These elements, not coming from the environment of origin, were considered as contaminants, and counted as negative (denoted as *contaminants*). In this case, the rarity of the element, so their presence in one or a few different skin samples, was considered a negative factor because the more ubiquitous the contaminant is, the less misleading it will be for future interpretation [27].

## Results

The analysis performed directly on the two positive control graphite stubs for the different environments, without including the skin factor, was used to reconstruct the characteristic debris of the tested environments from a morphological and chemical point of view.

### Characteristic elements of each environment

Based on these results, characteristic elements on the graphite control of each environment were identified (Table 1). The column on the right shows the total score of each environment obtained from the sum of the individual elements' scores.

**Table 1:** Elements and score of each of the six investigated environments obtained from the graphite control sample. The name “aggregate” has been used to identify an irregularly shaped collection of elements, while the term “chips” has been used to define thin curled strip objects

SITE	morphological elements	tot	chemical elements	tot
Florist	synthetic fibers, pollen, spores, diatoms, aggregate of particles, undetermined chips, algae, other botanical elements	34	Fe, Si, S, Al, Ca, Mg, Na, Au, K, Cu, Cr	39
Bakery	round particles single or aggregates (white particles), organic material	8	Na, Cl	6
Carpentry	wood chips, aggregates and single plant conduction tissues, soil, aggregate of particles	14	Ca, Si, Al, Na, Cl	13
Turning factory	metal chips, single and aggregate of particles	6	Fe, Cr, Au	12
Trunk	synthetic fibers, aggregate of particles, organic material, synthetic material	13	Cl, Ca, K, Si, Al	14
Construction site	single and aggregate of grey particles, organic material, soil	12	Ca, Si, Mg, S	12

Table 2 shows the score of each element based on the ubiquity or exclusivity of each.

**Table 2:** Proper elements recovered in the graphite control samples on the left; contaminants, recovered in the samples but not in the control, on the right

PROPER ELEMENTS				CONTAMINANTS			
morphological elements	value	chemical elements	value	morphological elements	value	chemical elements	value
		Fe	4	fiber (hair)	3	Fe	2
synthetic fibers	4	Ca	2	fiber	0	Ca	4
				dark fragment	0	P	5
round particles single or aggregates	5	Mg	4	pink fragment/light blue fragment	4	Mg	2
chips	5	Si	2	metal fragment	4	Si	4
metal chips	5			metal chips	5	Ti	5
organic material	3	Cu	5	organic material	2	Cu	4
wood chips	5			leaf fragment	4	Zn	3
plant stems	4			plant stem	4	Pb	5
botanical elements	4	K	4	botanical element	4	K	3
pollen/spore	5			spore	5	Ni	5
diatom	5	Al	3	fungal hyphae	5	Al	4
algae	5			bacteria	5	Ba	4
soil	4	S	4	soil	3	S	5
synthetic material	5			resin or glue	5	Sn	5
aggregates and single particles	1	Na	3	aggregates and single particles	2	Na	4
aggregates and single grey particles	5	Cr	4			Cr	5
		Au	4				
		Cl	3				

### Result of the analysis carried out on the skin samples

The results of the analyses carried out using the various techniques on the skin samples are outlined in the following paragraphs:

### *Naked eye observation—*Table 3, Fig. 1

**Table 3:**
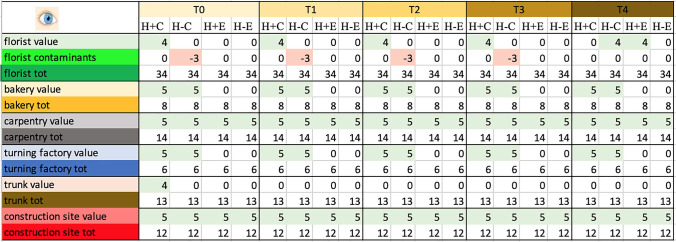
Total score, obtained by naked eye analysis, for the proper elements and for the contaminating elements of each sample and total score related to the environment in which the sample was placed

Symbols legend: H +  = hairy skin square; H- = Hairless skin square;  = put in contact; E = exposed

**Fig. 1:**
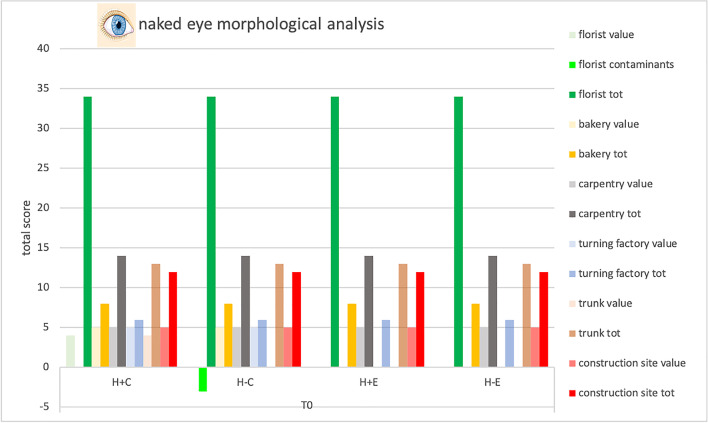
Total score, obtained at Time 0 by naked eye analysis, for the proper elements and for the contaminating elements of each sample and total score related to the environment in which the sample was placed. Symbols legend: H +  = hairy skin square; H- = Hairless skin square; C = put in contact; E = exposed

#### Debris on negative control

Negative skin control samples did not exhibit any particulate on their surface.

#### Debris rate on contact versus exposed samples

More than 90% of skin samples made to adhere to surfaces had macroscopically visible elements at T0, over 80% at T1, T2, T3, frequency decreasing to 75% at T4.

Samples in which the skin surface analyzed did not come into direct contact with the environment showed macroscopic particulate in about 30% of cases (T0, T1, T2, T3).

Carpenter and construction site were the only two environments whose samples did not show differences between contact and exposed samples.

#### Debris rate on hair and hairless samples

The presence and absence of hair did not result in major differences in terms of visible particulate, only samples from the florist show particulate matter predominantly distributed on the hairy skin.

#### Debris on skin samples from the six environments

The florist environment, one of the richest in elements, only reached a maximum of 4 points from the hair skin sample placed in direct contact out of 34 from the positive control; only fibers were found, an element proper to the environment but not exclusive. The baker obtained 5 points from the contact sample out of 8 from the positive control; in fact, abundant white powder exclusive of such an environment was observed. The case of the carpenter was similar, where the samples showed abundant wood chips, exclusive of this environment. Samples from the trunk show fibers only at time 0 and only from the hair contact sample, element proper but not exclusive of this environment. Finally, the construction site scores 5 out of 12 points, all samples in fact showing gray-colored particulate matter, an element exclusive to the environment.

Except for the hair found in the florist's sample, all the other elements recovered in the samples were consistent with those found in the control samples of the relative environments.

#### Qualitative effect of time on samples and particulate

Environmental trace materials did seem not to have suffered any qualitative alteration over time. The skin samples undergo two macroscopic modifications: centripetal desiccation and the appearance of a superficial greasy film.

### *Episcopic microscope observation—*Table 4, Fig. 2

**Table 4:**
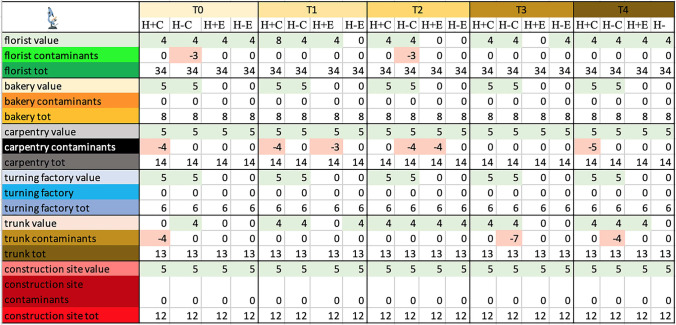
Total score, obtained by episcopic analysis, for the proper elements and for the contaminating elements of each sample and total score related to the environment in which the sample was placed

Symbols legend: H +  = hairy skin square; H- = Hairless skin square; C = put in contact; E = exposed

**Fig. 2:**
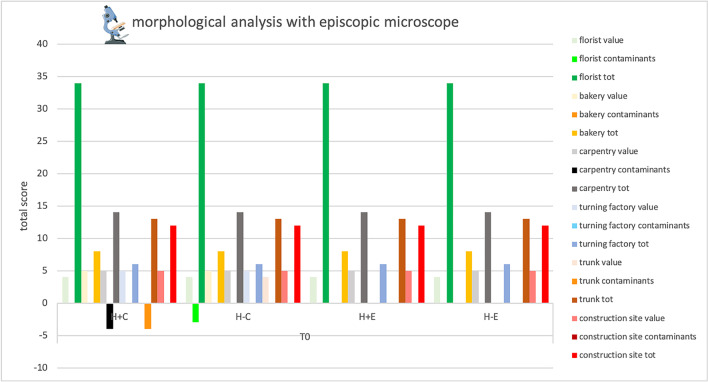
Total score, obtained at Time 0 by episcopic analysis, for the proper elements and for the contaminating elements of each sample and total score related to the environment in which the sample was placed. Symbols legend: H +  = hairy skin square; H- = Hairless skin square; C = put in contact; E = exposed

#### Debris on negative control

Negative skin control samples did not exhibit any particulate on their surface.

#### Debris rate on contact versus exposed samples

All the contact samples had visible elements from T0 to T4, except the hairy samples from the trunk and the florist environment, appearing clean.

Samples that were only exposed to the different environments showed proper elements in over 90% of cases at T0, the percentage decreasing in subsequent times up to 60%. Carpenter and construction site are the only two environments whose samples did not show differences between contact and exposure samples.

#### Debris rate on hair and hairless samples

The presence and absence of hair did not result in major differences in terms of visible particulate.

#### Debris on skin samples from the six environments

Proper elements, visible with the naked eyes are still visible with the episcopic microscope and better characterized, however, a great number of samples showed new small elements visible under episcopic microscopy.With the present technique, as well as more of its own elements, many non-proper elements were observed in samples from florists, carpentry, and trunk, starting from T0 (hair, dark fragment, and fibers). These improper elements were not exclusive to a single environment in all cases, as indicated by the scores.

#### Qualitative effect of time on samples and particulate

Qualitative features of particulate appear to be preserved over time. Centripetal desiccation of the skin squares, and appearance of a superficial greasy film were confirmed with this technique.

### *Double direct ED-XRF study-* Table 5, Fig. 3

**Table 5:**
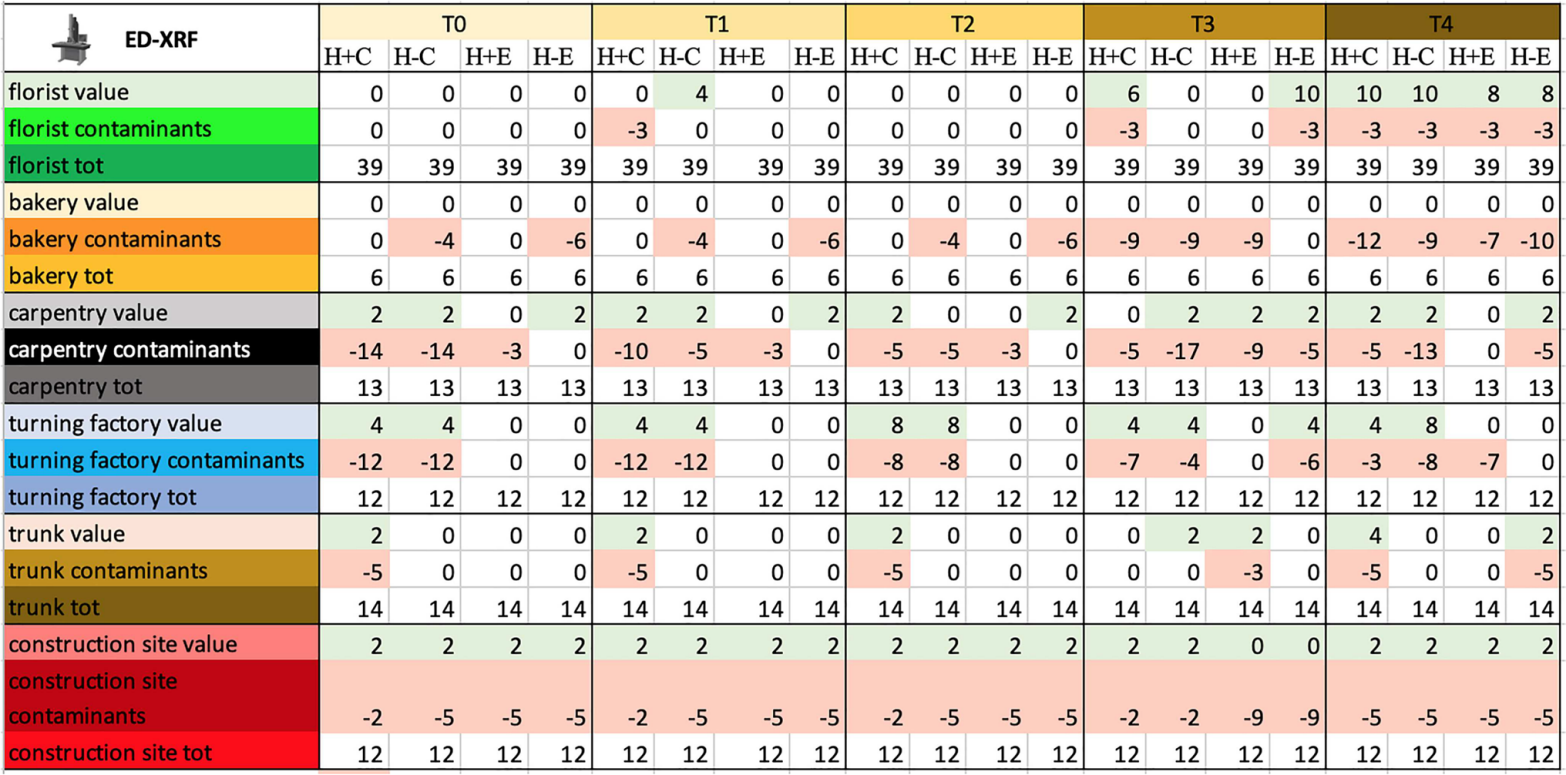
Total score, obtained by ED-XRF analysis, for the proper elements and for the contaminating elements of each sample and total score related to the environment in which the sample was placed

Symbols legend: H +  = hairy skin square; H- = Hairless skin square; C = put in contact; E = exposed

**Fig. 3:**
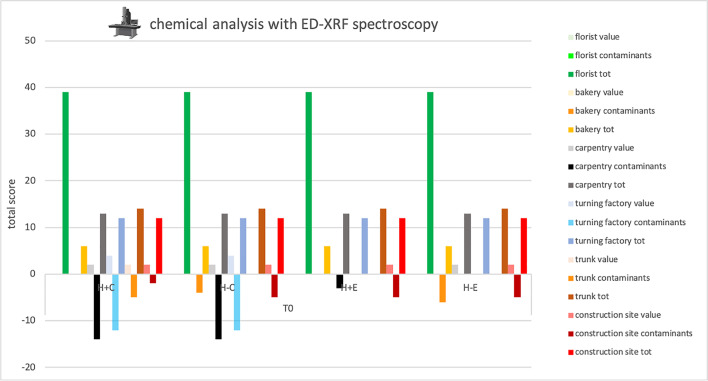
Total score, obtained at Time 0 by ED-XRF analysis, for the proper elements and for the contaminating elements of each sample and total score related to the environment in which the sample was placed. Symbols legend: H +  = hairy skin square; H- = Hairless skin square; C = put in contact; E = exposed

#### Debris on negative control

Negative skin controls were found to be free of chemical elements.

#### Debris rate on contact versus exposed samples

The contact samples generally had more elements than exposed ones, both proper (58% vs. 25%) and improper (67% vs. 33%), at time 0. However, at successive experimental times, the appearance of new elements, both proper and improper, on the samples can be observed. In fact, at time 4, 58% of the contact samples show proper elements versus 33% of the exposed ones, and 92% of the contact samples show improper elements versus 75% of the exposed ones.

#### Debris rate on hair and hairless samples

The presence and absence of hair did not result in major differences in terms of visible particulate.

#### Debris on skin samples from the six environments

Many samples, did not show any element on it; this is particularly evident on samples from florists and bakery, both characterized by organic materials, that cannot be detected with ED-XRF.

Elements, when present, were scarce and often unspecific, as evidenced by the low scores in Table 5. The only exception was represented by samples from the turning factory, in these samples iron was almost always present on contact samples. In contrast, contaminants were abundant and their score often exceeded that of proper elements, particularly in carpentry, construction site, and turning factory.

#### Qualitative effect of time on samples and particulate

ED-XRF did not identify any significant change in the contaminants at different time intervals.

### *SEM–EDX study*—Table 6, Fig. 4

**Table 6:**
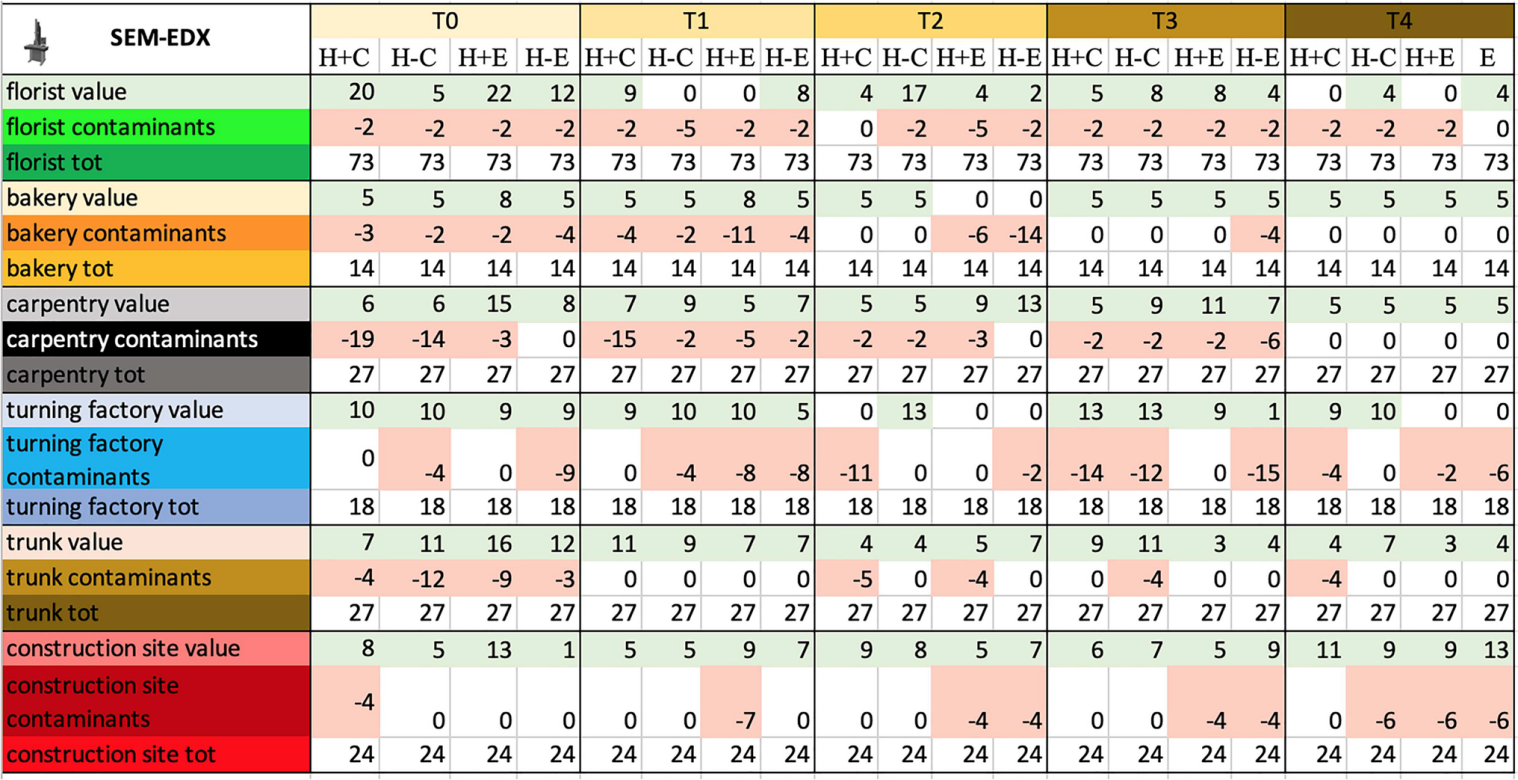
Total score, obtained by SEM-EDX analysis, for the proper elements and for the contaminating elements of each sample and total score related to the environment in which the sample was placed

Symbols legend: H +  = hairy skin square; H- = Hairless skin square; C = put in contact; E = exposed

**Fig. 4:**
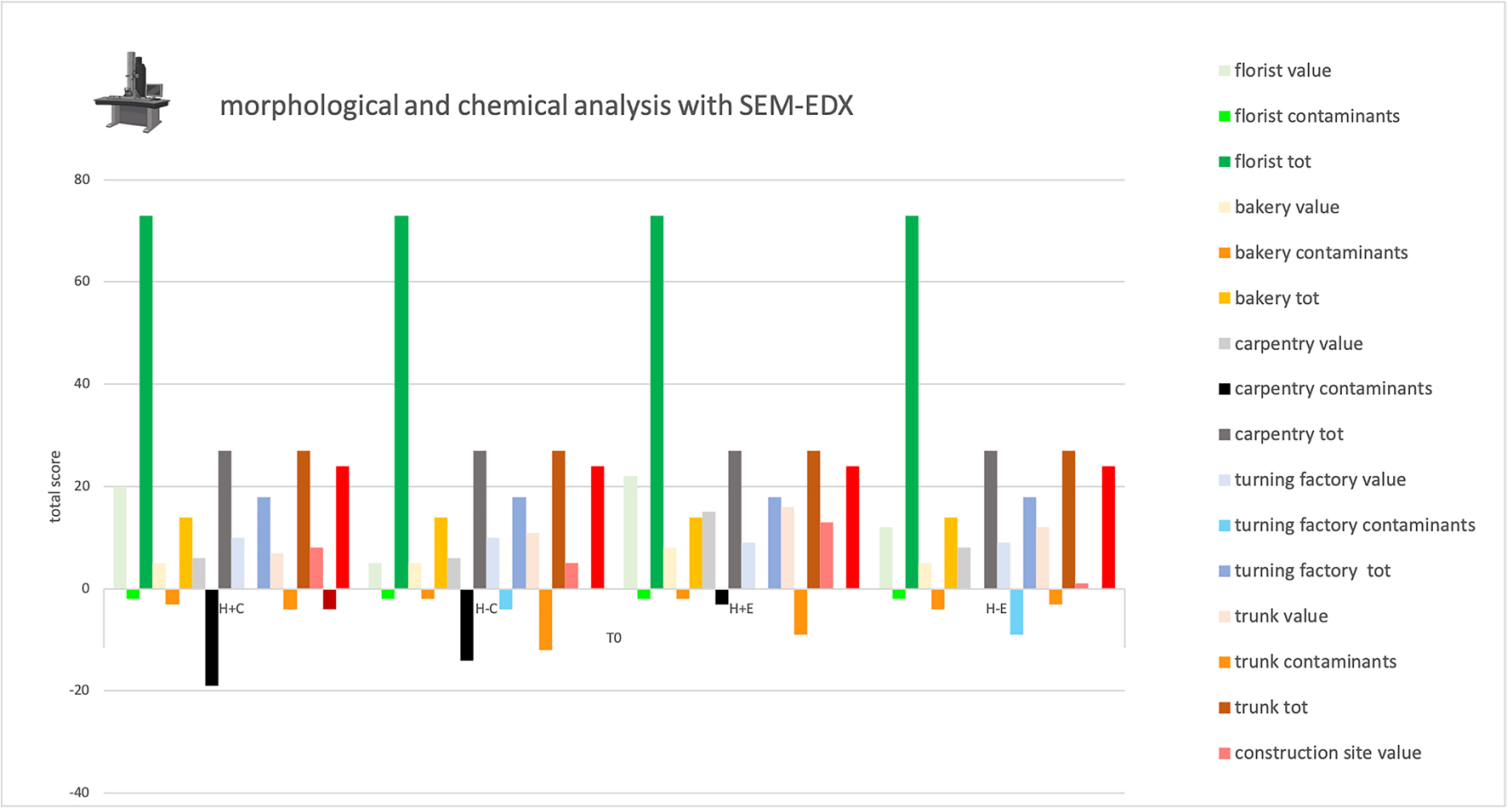
Total score, obtained at Time 0 by SEM–EDX analysis, for the proper elements and for the contaminating elements of each sample and total score related to the environment in which the sample was placed. Symbols legend: H +  = hairy skin square; H- = Hairless skin square; C = put in contact; E = exposed

#### Debris on negative control

Morphological examination performed on negative skin controls revealed the presence of organic and botanical material, both on hairy and hairless samples. The chemical analysis (EDX), on the other hand, did not detect any inorganic particulate.

#### Debris rate on contact versus exposed samples

All the contact samples as well as samples that were only exposed in carpentry, and construction site, showed particulate from T0 to T4. In the remaining environments, samples with particulate matter were still over 75%.

#### Debris rate on hair and hairless samples

The presence and absence of hair did not result in major differences in terms of visible particulate.

#### Debris on skin samples from the six environments

Own elements were abundant and specific in almost all samples, only in 17% of the samples, the score of the contaminants exceeded that of the own elements.

#### Qualitative effect of time on samples and particulate

SEM–EDX did not identify any significant qualitative change of particulate at different time intervals.

Tables showing the integral results obtained with the different analysis techniques can be accessed as supplementary material.

### Synthesis: focus on proper element and contaminant scores

**Fig. 5:**
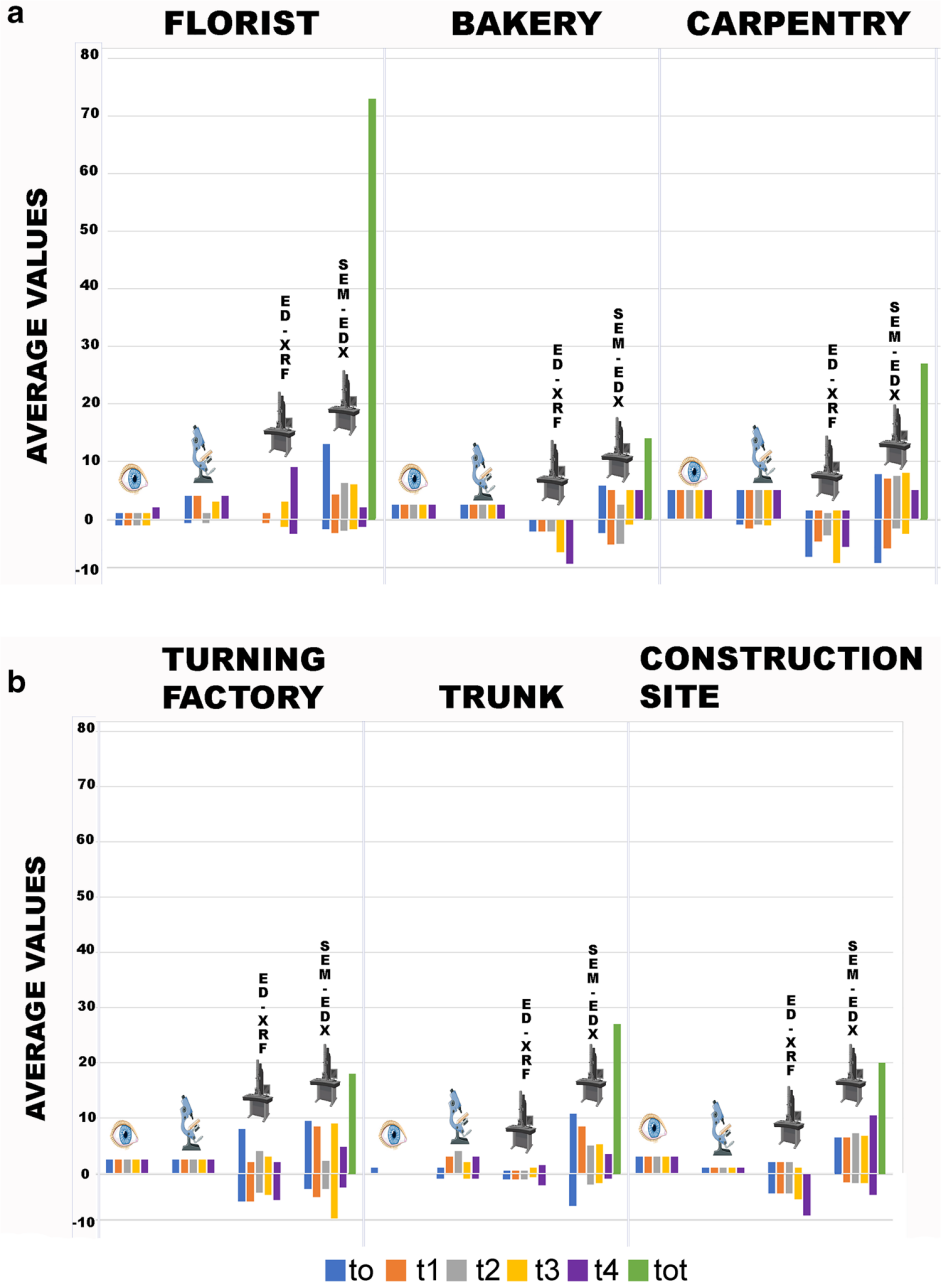
**A, B** Summary histogram of the average values obtained from the 4 samples (with hair, without hair, in contact, and exposed) of proper elements (positive value) and contaminants (negative value) for each time observed with each of the four survey techniques. In green the total value obtained from each environment (sum of the scores of all elements observed on the control samples placed in the same environment)

The histogram below (Fig. 5) summarized the average values obtained from the 4 samples (with hair, without hair, in contact and exposed) of proper elements and contaminants for each time observed with each of the four survey techniques, compared with the total value obtained from each environment as the sum of the scores of all elements observed on the control samples placed in the same environment.

As for the proper elements, the analysis with the naked eye and that with the episcopic microscope had the same score in four of the six environments analyzed, in the remaining two, however, the score increases with the observation at greater magnification. The XRF analysis, not comparable to the previous techniques as it only provides a chemical analysis, gave extremely low scores in five of the six environments while giving higher values in the turnery. Finally, the SEM–EDX analysis resulted in the highest scores for all environments.

Considering the contaminants, the analysis with the naked eye and that in episcopic microscopy provided similar results, even the greater magnification provided by microscopy allowed in some cases to observe widespread or even ubiquitous contaminants such as fibers of dark color (value 0 as present in 6 out of 6 environments), not observed with the naked eye. XRF analysis showed high contaminant values, when compared to the values of identified own elements, in three of the six environments: carpentry, turnery, and construction site. Finally, the SEM–EDX analysis showed contaminant values similar to or lower than those obtained from the proper element analysis in all environments. A clear predominance of own elements over contaminants was visible in florist, trunk and construction site. Differences between ED-XRF and SEM–EDX are linked to the different sensitivity for the various chemical elements; this aspect should be taken into account when choosing the analytical technique to be applied for debris detection.

## Discussion

With the present study, we have experimentally recreated the interaction between skin and environment to test and compare four conservative methods of analysis of environmental debris. The goal was to investigate the possibility and best way to obtain information about a violent crime victim's background from microscopic residues on the skin. The need arose from certain homicide cases that were dealt with in recent years by some of the authors [8]: on that occasion, it emerged how little literature there is on the subject. The following pages will discuss four main aspects of the experiment:

- effectiveness of analysis techniques;
- effect of dwelling environment, of exposure type and of possible presence of hair
- effect of time on samples and particulate

### Effectiveness of analysis techniques

Considering the number of samples showing macroscopically visible particulate matter, it seems clear how the simple but careful observation with the naked eye can guide the forensic scientist in identifying environmental particulate, whose nature, if not directly recognizable, can then be investigated more in depth with other methods of investigation [28]. However, it was possible to observe that most of the macroscopically visible debris was located on samples that had come into direct contact with the environment and much more rarely on those that had been exposed; it is, therefore, possible to infer that the simple exposure is not always enough to leave traces detectable to the naked eye.

Moreover, not all particles are macroscopically evident; therefore, the forensic scientist must use other methods of investigation, which allow to appreciate also the less visible elements of the debris.

**Fig. 6:**
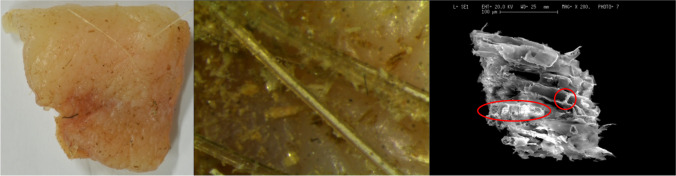
Image of the skin sample placed in contact with carpentry environment and the debris present on it. The debris is observed through the episcopic microscope (16X) (in the middle) and SEM (on the right). The image on the right shows a micrometric wood fragment, which retains identifying characteristics such as rays and vessels (circled in red) that could be used by specialized personnel for specific identification

In this regard, the use of the episcopic microscope has allowed not only to increase the number of samples on which debris is appreciable, but also to obtain a more detailed morphological characterization of particles. This, in turn, has made it possible to define in more specific terms some elements present in the samples and thus to assign them a higher specificity score (e.g. passing from botanical material to algae). Therefore, the involvement of specific forensic specialists, already at this stage of the investigation, could allow maximizing the information obtainable. This situation was then further amplified with the use of XRF and SEM–EDX (Fig. 6), which have added additional and more complex information (eg. in addition to morphology, the chemical composition of the elements present on the sample was added), immediately emphasizing the need to involve further experts.

The use of XRF makes it possible to analyze all the chemical components present on a surface of 0.5 cm^2^, without however being able to trace organic matrices and the chemical compounds to which the single elements detected belong to. The importance of this investigation is linked to the possibility of infinitely repeat the individual analysis (each lasting a few seconds), without altering the sample.

Comparing the chemical investigations carried out by SEM–EDX with those obtained by XRF, the two methods are nearly overlapping in the identification of all chemical elements, except, those with atomic number less than 19 which XRF, unlike SEM–EDX, does not detect. This limitation justifies the low effectiveness of this technique on samples such as those from the bakery, carpentry and construction site, which, deprived of the most characteristic light elements, present only common elements such as calcium.

Hence the importance, for further investigation, of the use of SEM–EDX that allows investigating in an extremely accurate way the morphology of the contaminated, to obtain the dimensions and chemical composition.

This investigation, however, requires that the sample is treated in a particular way (metalized), implying a non-reversible operation on the samples. Its application should be the final step of investigation. However, there are new types of SEM, such as E-SEM [29], that allow analyzing in their natural state even "humid" samples and especially non-conductive samples, so that they can be observed in their natural state in low vacuum, without the need for conductive coating (metallization).

Both techniques show limitations in terms of chemical matrix of the traces and therefore also concerning answers to questions raised by the specific case, so it will be desirable that future studies could benefit from the use of a dedicated tool such as the FT-IR spectroscopy, a already widely used in the forensic context for comparative and identification analysis on materials of various nature [30–32]. This technology could help in demonstrating elements and compounds more specific or even exclusive of a certain environment.

### Effect of dwelling environment, of exposure type and of possible presence of hair

**Fig. 7:**
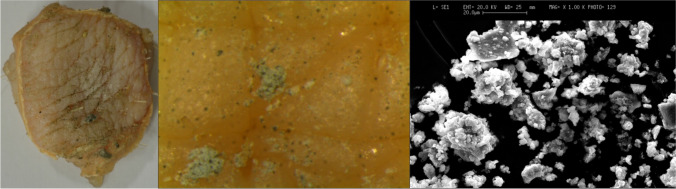
Image of the skin sample placed in contact with the environment of the construction site and the particulate present on it. The debris is observed through the episcopic microscope (16X) (in the middle) and SEM (on the right)

**Fig. 8:**
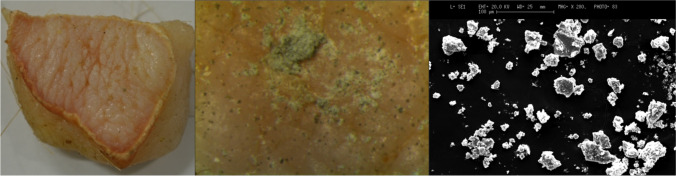
Image of the skin sample placed exposed in the construction site and the particulate present on it. The debris is observed through the episcopic microscope (16X) (in the middle) and SEM (on the right)

Contaminated samples were quite more numerous among contact samples than the exposed ones; this difference is particularly evident on the samples when observed with the naked eye, while it appears less marked when using more thorough investigation techniques. So, the direct contact between body and environment seems to be an important element for the finding of macroscopically visible particulate matter, even if it is not always necessary, especially as concerns operations that tend to disperse large amounts of material are taking place, e.g. the phase of mixing flour products in the bakery or that of mixing and handling lime and cement in the construction site (Fig. 7 and 8).

Concerning the presence of hair, it does not seem to significantly affect the retention of particulate.

Moving on to consider the different environments selected, it was possible to identify particulate, albeit with different degrees of specificity, typical of each environment, both from the morphological and chemical points of view. For example, we observed the presence of wood chips in carpentry, metal chips in the turnery, white powder, probably flour, in the bakery, botanical elements, such as algae in the florist, synthetic fibers in the florist and the trunk of the car.

Considering contaminants, interesting is the ubiquitous presence of fibers, as already reported in literature [33–37], even though, as previously reported, synthetic fibers are characteristic of only two environments, florist and car trunk.

In addition, some chemicals were observed to be more represented in certain environments (e.g. Fe and Cr in the turnery), while others are present almost ubiquitously and are therefore poorly characterized [27]. The chemical analyses carried out have shown that there is often a quite ubiquitous (4 out of 6 environments) presence in the environments of calcium and silicon, elements whose presence alone cannot, therefore, be considered useful for environmental characterization. However, their presence, associated with less common elements, such as magnesium (2 out of 6 environments), can be characteristic, is the case of the construction site. In this context, the inability for an analysis such as XRF spectroscopy, to determine light elements such as Mg, elements potentially characterizing a given environment, can be extremely limiting.

However, at a semi-quantitative level, a greater presence of calcium in the form of calcite/gypsum was recorded in the construction site, which can be explained by the use of skimming and plastering products in that environment.

It was also noted that environments where the organic component of particulate prevails (for example, the florist and the bakery), as well as a closed environment such as the car, in which no particular activity takes place, are less characterizable for debris, both from a morphological and chemical point of view.

### Effect of time on samples and particulate

Finally, considering the effect of time on skin samples and debris, it was observed that the particulate present on the skin samples have maintained their morphological and chemical characteristic over time (from 0 to 30 days after contact or exposition to the environment). However, it must be taken into account that the samples were conserved in a protected environment. Further studies could evaluate instead, the preservation of debris by placing the samples in an open environment, therefore more exposed to various external factors.

The tested substrate, skin, also seems to maintain its morphological characteristics for the entire trial, retaining almost unchanged its evidence-trapping properties.

In addition, only when all analyses are conducted on the same sample is it possible and reasonable to make a quantitative analysis of the particulate matter present. Here the destructive nature of some techniques and the small number of samples did not give this possibility, but subsequent studies on a larger number of samples or made with non-destructive techniques could allow associating to the qualitative piece of information also a quantitative one.

## Conclusions

The external examination of the corpse is of great significance to the investigations both during the crime scene inspection and the autopsy. In this phase, it is possible to collect traces useful to identify not only the subjects and objects involved in a criminal event but also the places where it took place.

Direct contact of the victim's skin with the environment, as opposed to just exposing it in a given environment without direct contact, seems to be an important element for the finding of macroscopically visible particulate matter, even if direct contact is not always necessary, especially if operations that tend to disperse material are taking place i.e., in the bakery or the construction site. This turns out to be an aspect of considerable interest in case of, for example, a body left at a construction site but inside an open suitcase or in a box, or partially protected by a carpet and thus not in direct contact with the floor of the site itself.

Skin seems to be able to retain environmental traces up to a month after the interaction, and traces may be detectable even with only naked-eye observation. However, the simple observation with the naked eye of such small and fragmented elements is not sufficient by itself to be able to characterize them so specifically as to derive useful information and must be accompanied by detailed morphological and chemical analyses (e.g., what was determined as a plant fragment by the naked eye might turn out to be an ivy trichome under microscopy, allowing a much more detailed and identifying characteristics of the environment of origin). The investigations must be further detailed with methods that detect even smaller size particles and characterize them morphologically and chemically, thus providing the forensic scientist with a more complete picture, even though it will be more complex to interpret. In this regard, an aid to interpretation could come from the creation of databases that collect particles/elements typical of specific environments, starting with those most characteristic in terms of type and abundance of particulate matter present, such as some of the workplaces tested in this article. Then, this tool could automatically compare the sample with the environments in the database, assigning a compatibility value that could direct investigators.

In conclusion, it is possible to detect numerous traces on a victim’s corpse, even in the clean skin areas, up to a month after exposure/contact. Chemical and thorough morphological analyses are useful to further characterize the debris, but the interpretation of their results demands the cooperation of different forensic science specialties, to properly enhance the value that every trace carries within itself.

### Supplementary Information

Below is the link to the electronic supplementary material.

Supplementary file1 (DOCX 1104 KB)

## Data Availability

All data are available in the article or supplementary material.
