# Supplementary material for: Skin surface debris as an archive of environmental traces: an investigation through the naked eye, episcopic microscope, ED-XRF, and SEM–EDX
Source: Int J Legal Med. 2023 May 25;138(1):123–37. doi: 10.1007/s00414-023-03021-1 (PMC10772008; doi:10.1007/s00414-023-03021-1)

*Supplementary material*

Below are reported tables of observations made on samples from the different environments at different experimental times. In green are the proper elements, those also present in the control samples, in black those not present in the control samples and therefore considered contaminants.

*Naked eye observation*

*
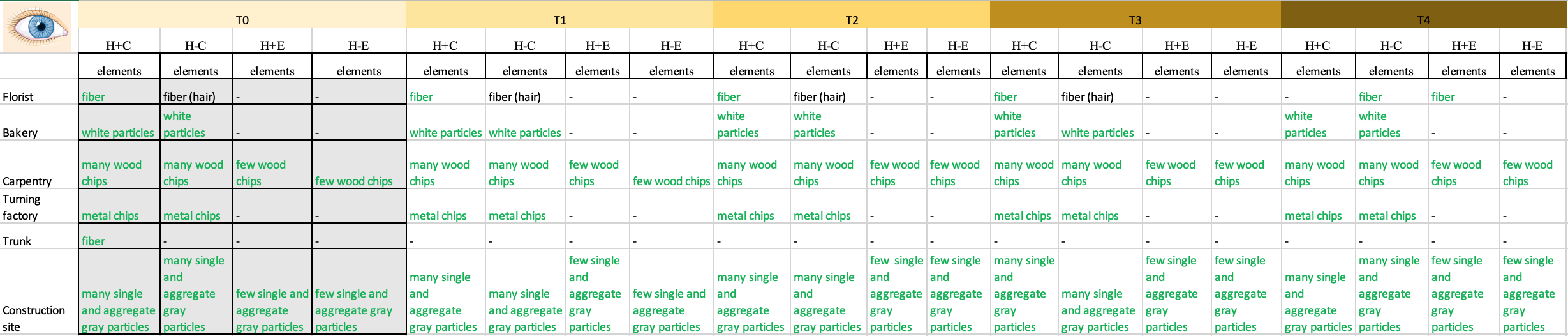
*

*Episcopic microscope observation*


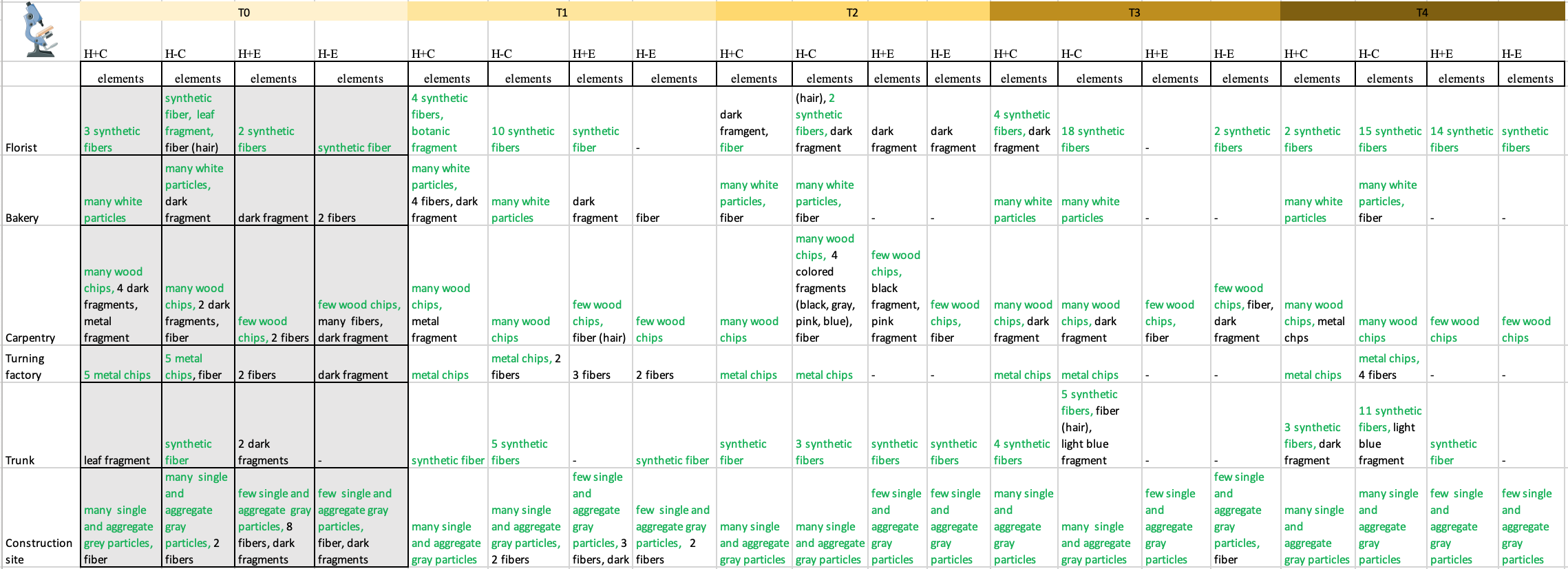


*Double direct ED-XRF study on the upper half*


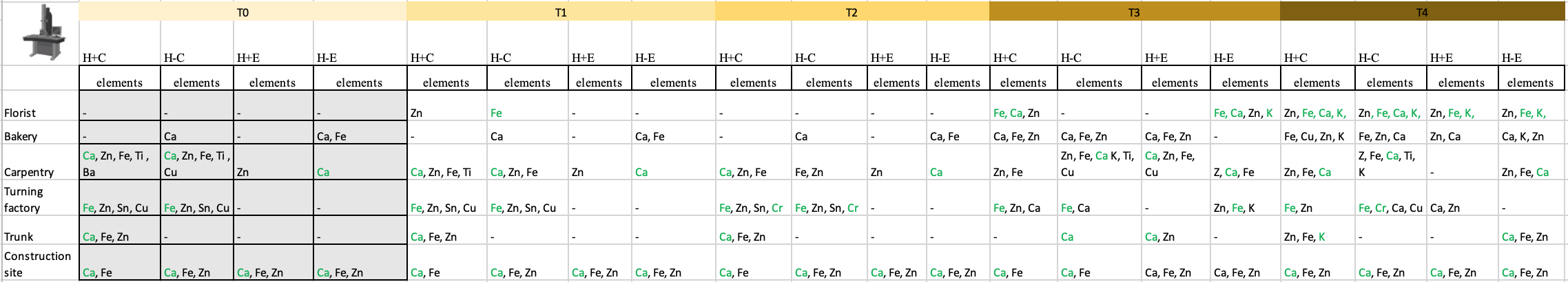


*SEM-EDX study of the lower half*


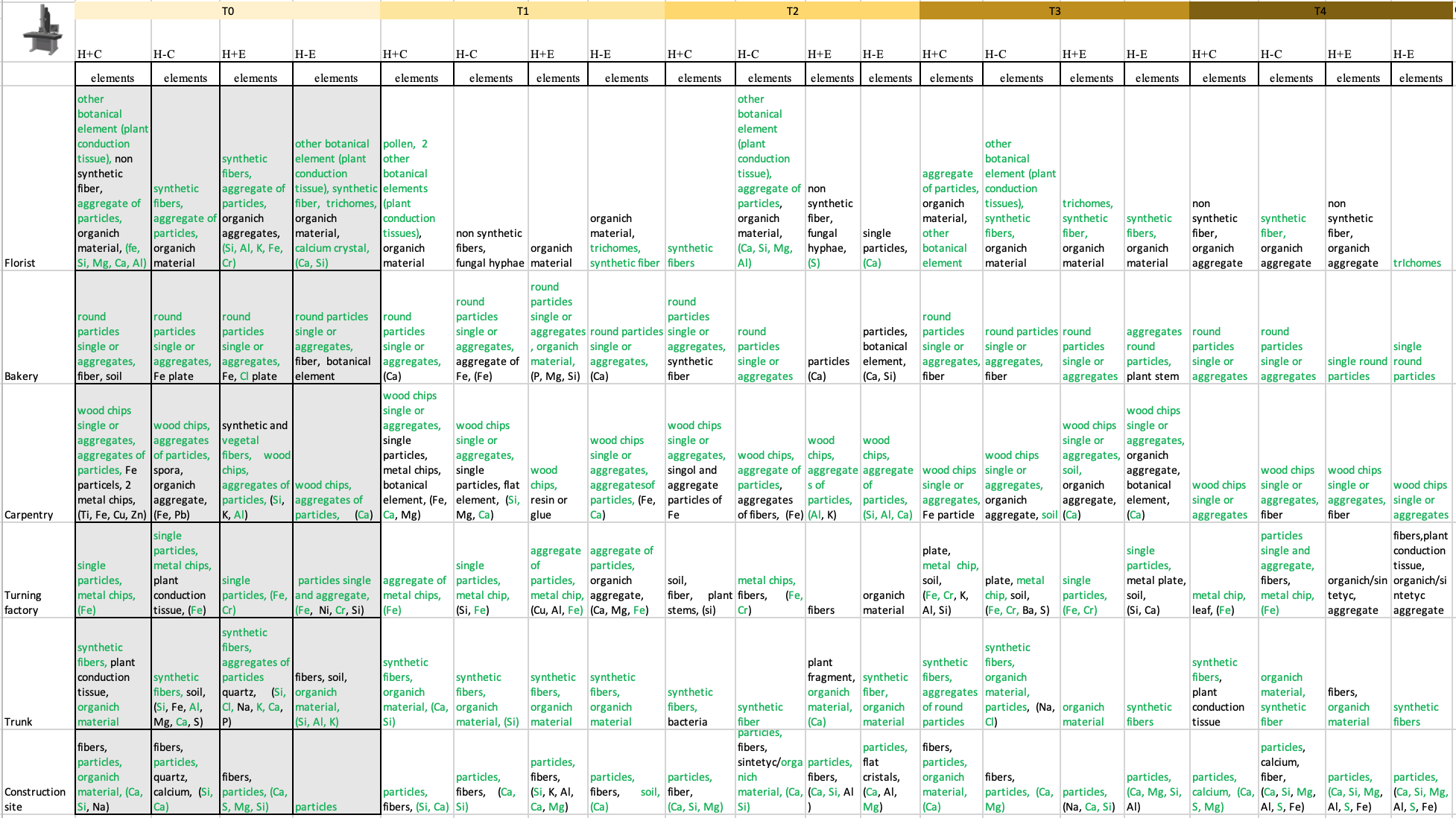

Supplement: Supplementary file 1 — Supplementary file1 (DOCX 1104 KB) [file 414_2023_3021_MOESM1_ESM.docx]
